# Make care coordination a high priority

**DOI:** 10.1177/2053434517745192

**Published:** 2017-12-14

**Authors:** Hubertus JM Vrijhoef

**Affiliations:** 1 Maastricht University Medical Center, the Netherlands; 2 Panaxea b.v., the Netherlands; 3 Vrije Universiteit Brussels, Belgium

According to the International Experts Working Group on Patients with Complex Needs, established by the Commonwealth Fund, the first out of 10 prerequisites of a high-performing healthcare system for people with multiple health problems is to make care coordination a high priority.^1^ The other nine recommendations are:

- identify patients in greatest need of proactive, coordinated care (#2);
- train more primary care physicians and geriatricians (#3);
- facilitate communication between providers (#4);
- engage patients in decisions about their care (#5);
- provide better support for caregivers (#6);
- redesign funding mechanisms to meet patients’ needs (#7);
- integrate health and social services, and physical and mental healthcare (#8);
- engage clinicians in change and train and support clinical leaders (#9); and
- learn from experience and scale up successful projects (#10).^1^

The Group notifies that “all 10 present challenges, with some requiring profound paradigm shifts.”^1^ Examples of such shifts are: moving away from disease-specific care delivery and toward person-centred approaches, or mowing away from the single-provider model and toward cooperation and teamwork.^1^ Further, the Group notifies that “their implementation has the potential to transform care and quality of life for millions.”^1^

The *International Journal of Care Coordination* is a unique source for those interested in reading about how to deal with these challenges and/or what their (potential) impact is. For example, in this issue, Samus et al. present a study proposal for evaluation of a home-based care coordination model for people with dementia, which is now being tested in a demonstration funded by the US Center for Medicare and Medicaid Innovation (recommendation #1).^2^

Grustam et al. describe the differences in care coordination and transaction costs between a business-to-business model and a business-to-consumer model for tele-monitoring applications for patients with chronic conditions (recommendation #7).^3^ Hall et al. compared the impact of an emergency department-to-home coaching intervention to usual, post-emergency department care on multiple patient-reported health-related quality-of-life measures (recommendation #8).^5^

Adjemian et al. performed a descriptive systematic review to examine the proportion of clinical pathway publications in an emergency department setting that adequately reported: (1) the exact reproduction of the clinical pathway that was implemented in the study, (2) the adherence to and correct execution of the clinical pathway intervention and (3) the presence of a pre-implementation education phase (recommendation #9).^4^ Baldewijns et al. present a study proposal for the INTERACT-in-HF study aiming to explore the current processes of Heart Failure care and to identify barriers and facilitators for improvement of Heart Failure care and guideline adherence in three regions in the North-West of Europe (recommendation #10).^6^

Interestingly, the five papers in this issue each seem to deal with a single prerequisite of a high-performing healthcare system, as identified by the International Experts Working Group on Patients with Complex Needs.^1^ The next step then is to comprehensively address multiple recommendations and challenges. Most likely, our next special issue on realist evaluation, scheduled for Spring 2018, will shed a light on how to address the interrelatedness of context, mechanisms and outcomes of recommended strategies to improve care coordination.^7^ The call for submission is open till 31 December 2017.
